# Differential diagnosis of bacterial from candidal bloodstream infections in ICU patients: the role of procalcitonin

**DOI:** 10.1186/cc14148

**Published:** 2015-03-16

**Authors:** E Angelopoulos, E Perivolioti, S Kokkoris, E Douka, E Barbouti, P Temperekidis, C Vrettou, C Psachoulia, G Poulakou, S Zakynthinos, C Routsi

**Affiliations:** 1 Evangelismos Hospital, Athens, Greece; 2 Attikon Hospital, Athens, Greece

## Introduction

Early differentiation of bacterial from candidal bloodstream infections (BSIs) in the presence of sepsis or septic shock is crucial because of the need for appropriate treatment initiation. Clinical data, although limited, suggest a role for procalcitonin (PCT) [1-3]. The aim of this study was to investigate a possible association between the etiology of BSIs and the serum PCT levels.

## Methods

ICU patients with clinical and laboratory signs of sepsis or septic shock, with documented BSIs and with both serum PCT and CRP measurements on the day of the positive blood sample (±1 day), were included. Illness severity was assessed by SOFA score on both admission and BSI day. Demographic, clinical, and laboratory data including PCT and CRP levels, as well as the white blood cell (WBC) count on the day of the BSI were recorded. PCT was measured by an electrochemiluminescence analyzer and CRP by the tholosimetric method (Roche, Switzerland).

## Results

A total of 64 ICU patients (mean age 58 ± 18 years, 39 males) with BSIs were included. SOFA sore was 9 ± 4 on ICU admission and 8 ± 4 on the day of BSI. In 30 of these patients *Candida *spp. were isolated in blood culture (candidemia group) whereas the remaining 34 had a bacterial etiology of BSI (bacteremia group). Serum PCT concentrations remained within normal ranges in most patients with candidemia whereas a wide range was observed in patients with bacteremia. Mean values of PCT and CRP levels were higher in the bacterial than in the candidemia BSI group: 18.5 ± 33.2 versus 0.73 ± 1.40 ng/ml, *P *< 0.001 and 17.7 ± 10.3 versus 8.9 ± 8.0 mg/dl, *P *= 0.001, respectively. There was a significant difference in WBC count between the two groups: 19,460 ± 10.174 versus 11,000 ± 5,440, *P *< 0.001 for the bacteremia and candidemia BSI group, respectively. A ROC curve analysis of the predictive ability of PCT showed an AUC of 0.79 (*P *< 0.001). When a cutoff point of 0.40 ng/ml was selected using Youden's *J *statistic, a low value of PCT had in our sample a negative predictive value of 0.76 and a likelihood ratio (negative) of 0.30.\

## Conclusion

A low serum PCT value could be considered as a diagnostic marker in distinguishing between BSIs of candidal or bacterial origin in ICU patients with varying severity of sepsis.
